# A 23-dB bismuth-doped optical fiber amplifier for a 1700-nm band

**DOI:** 10.1038/srep28939

**Published:** 2016-06-30

**Authors:** Sergei V. Firstov, Sergey V. Alyshev, Konstantin E. Riumkin, Vladimir F. Khopin, Alexey N. Guryanov, Mikhail A. Melkumov, Evgeny M. Dianov

**Affiliations:** 1 Fiber Optics Research Center of the Russian Academy of Sciences, 38 Vavilov str., Moscow 119333, Russia; 2 Institute of Chemistry of High-Purity Substances of the Russian Academy of Sciences, 49 Tropinin str., Nizhny Novgorod, 603600, Russia

## Abstract

It is now almost twenty-five years since the first Erbium-Doped Fiber Amplifier (EDFA) was demonstrated. Currently, the EDFA is one of the most important elements widely used in different kinds of fiber-optic communication systems. However, driven by a constantly increasing demand, the network traffic, growing exponentially over decades, will lead to the overload of these systems (“capacity crunch”) because the operation of the EDFA is limited to a spectral region of 1530–1610 nm. It will require a search for new technologies and, in this respect, the development of optical amplifiers for new spectral regions can be a promising approach. Most of fiber-optic amplifiers are created using rare-earth-doped materials. As a result, wide bands in shorter (1150–1530 nm) and longer wavelength (1600–1750 nm) regions with respect to the gain band of Er-doped fibers are still uncovered. Here we report on the development of a novel fiber amplifier operating in a spectral region of 1640–1770 nm pumped by commercially available laser diodes at 1550 nm. This amplifier was realized using bismuth-doped high-germania silicate fibers fabricated by MCVD technique.

Bismuth-doped optical fibers are attractive active materials for various applications due to their unique optical properties. First bismuth-doped optical fibers with the core made of aluminosilicate glass were fabricated by Modified Chemical Vapor Deposition (MCVD) technique in 2005 1,2. Thereafter, this type of fibers was utilized to obtain lasing and amplification in the near IR region (1150–1215 nm)^3^,^4^,^5^,^6^,^7^. Further investigations showed that luminescence, absorption and gain spectra of bismuth-doped fibers strongly depend on chemical glass composition. It led to the development of Bi-doped fibers with different core glass compositions: silica, germanosilicate, phosphorosilicate etc^8^,^9^. A family of high-power continuous-wave and mode-locked lasers^10^,^11^,^12^,^13^, superfluorescent sources^14^,^15^ and optical amplifiers^16^,^17^ operating in a wavelength region of 1260–1550 nm was developed using Bi-doped fibers.

Recently, we demonstrated that bismuth-doped fibers can also be used as an active medium for a novel type of CW lasers radiating in a spectral region of 1625–1775 nm with the output power more than 1 W 18,19. At present, considerable attention is paid to a wavelength range of 1600 to 1900 nm lying between Er -doped and Tm -doped fiber gain bands because it has a potential to be a new transmission window for optical communications based on hollow-core photonic-bandgap fibers^20^,^21^. It is clear that in this case optical amplifiers will be required. Up to now, Tm-doped ZBLYAN^22^ and fluoride fiber amplifiers^23^ operating in this region have been created. In addition, the first demonstration of Tm-doped silica-based fiber amplifier (TDFA) operating in the 1650–1710 nm spectral range has also been reported^24^. However, all TDFAs are characterized by an intensive amplified spontaneous emission (ASE) in a wavelength region of 1800–1900 nm as a result of high population inversion of Tm ions. In order to obtain maximum gain around 1700 nm the ASE is depressed using special methods (such as Er-Tb co-doping, additional home-built ASE filter etc.) that is inconvenient for potential applications.

In this paper we report on the development of a novel Bismuth-Doped Fiber Amplifier (BDFA) for a spectral region of 1640–1770 nm which can be pumped by commercially available laser diodes. It is worth to note that 3-dB gain bandwidth of the developed BDFA is broader than other existing amplifiers operating in this region.

## Results and Discussion

We fabricated a family of single-mode fibers with a high content of germanium oxide in the silica-based glass core doped with a different Bi concentration. The fiber preforms with the core glass composition of Bi:50GeO_2_-50SiO_2_ were manufactured by the MCVD process. After additional jacketing fibers with the standard 125-μm cladding diameter, the ~2-μm core diameter, a cutoff wavelength of 1.2 μm and numerical aperture (NA) ~0.45 were drawn from these preform under common drawing conditions. Bismuth concentration in the preforms was controlled by means of the Electrothermal Atomization Atomic Absorption Spectrometry (EA-AAS), and the Inductively Coupled Plasma Atomic Emission Spectroscopy (ICP-AES). Detailed information on the investigated fibers is presented in Table 1.

**Figure 1:**
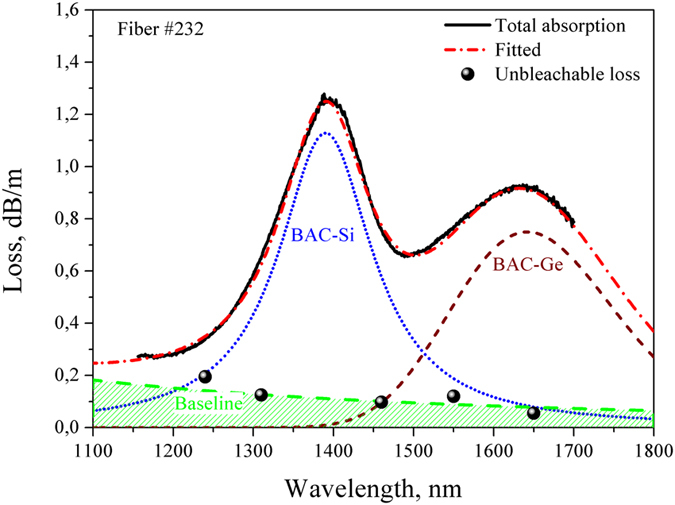
Absorption spectrum of Fiber #232. The shaded region indicates the estimated level of the unbleachable loss. The experimentally defined unbleachable loss level at the wavelength 1550 nm is shown by a ball-shaped point.

A typical absorption spectrum of this type of fibers (Fiber #232 from Table 1) is shown in Fig. 1. The obtained spectrum consists of two characteristic bands peaking at 1400 and 1650 nm. As it is known^25^,^26^,^27^ the first band in the spectrum belongs to the bismuth-related active centers associated with silicon (BACs-Si) whereas the second one is assigned to the bismuth-related active centers associated with germanium (BACs-Ge). These fibers can be used to obtain lasing at 1700 nm as well as at 1400 nm^18^. In the present paper we focus on the BAC-Ge because these centers are used to achieve gain in the band of 1700 nm. The absorption at the wavelength of 1650 nm without unbleachable loss can be used to estimate the concentration of BACs associated with Ge (Table 1). It should be noted that unbleachable loss as well as bands of BACs appear as a result of the incorporation of bismuth in fiber glass core. The unbleachable loss is the main reason for the decrease of the laser efficiency of bismuth-doped fibers. Fibers possessing a lower unbleachable loss usually demonstrate better efficiency in lasing and optical amplification (ceteris paribus). The unbleachable loss level in all fibers was determined by measuring the absorption (cut-back method) at high level of input signal (20 mW) at the wavelength 1550 nm. For Fiber #232 we performed similar measurements at additional wavelengths in the spectral region 1200–1700 nm. Taking into account the obtained results for the other wavelengths the unbleachable loss spectrum determined was illustrated by the shaded region in Fig. 1. It is seen that the unbleachable loss level weakly depends on the wavelength in the 1300–1700-nm region. Therefore, the value of unbleachable loss at 1550 nm can be used as a good estimation of the unbleachable loss level at 1650 nm for all fibers in Table 1.

**Figure 2:**
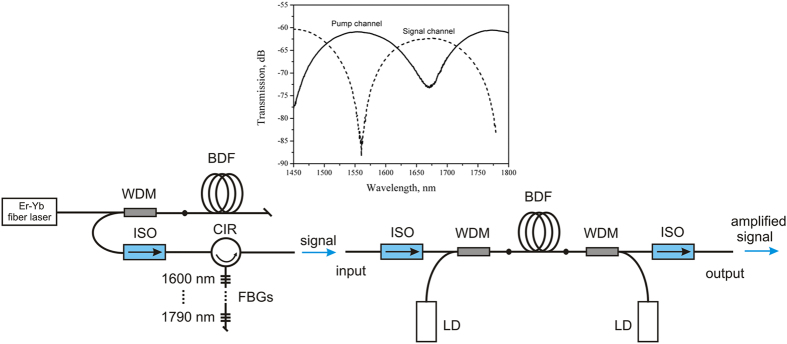
Experimental setup of BDFA. The left side of the figure shows a home-built light source generating wavelength comb starting from 1615 nm and ending at 1795 nm evenly spaced with the step of 15 nm. The right side of the figure demonstrates the amplifier itself. The abbreviations used: BDF – bismuth-doped fiber, WDM – wavelength division multiplexer (transmission spectra are illustrated at the top of the figure), ISO – optical isolator, LD – laser diode operating at 1550 nm, CIR – optical circulator, FBG – fiber Bragg grating.

The experimental setup of a typical Bi-doped fiber amplifier is illustrated schematically in Fig. 2. The BDFA was constructed using a scheme with bidirectional pumping (backward and forward pumping). As a pumping source we used commercially available laser diodes with a maximum output power of 150 mW each. The active fiber was core-pumped through commercially available WDMs based on SMF-28. Transmission spectra of the used WDMs are presented in Fig. 2. Optical isolators were spliced to the input and output of the amplifier. The first one was used to reduce the effect of amplified spontaneous emission (ASE) of a bismuth fiber on the signal source. The second one prevented possible lasing. Bold points represent splices where an optical loss was ~1 dB because of the difference between the mode field diameter of the active fiber and that of conventional fibers (SMF-28).

**Figure 3:**
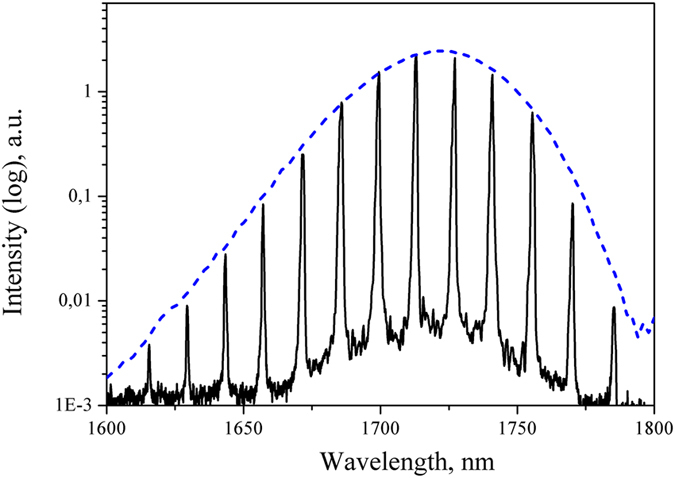
Spectra of a superluminescent bismuth-doped fiber source (dashed line) and the input signal (solid line). Spectral resolution is 0.2 nm.

To measure spectral dependencies of the gain and the noise figure of the amplifier a multi-line signal source was constructed using a bismuth-doped superluminescent source emitting in a wavelength region of 1600–1800 nm (Fig. 3) and a set of high-reflective fiber Bragg gratings (FBGs) in combination with an optical circulator (CIR) (Fig. 2, left).

The resulting input signal spectrum had the form of a spectral comb consisting of 13 narrow (about 1 nm) lines (Fig. 3). The lines were evenly spaced with 15 nm step starting from 1615 nm and ending at 1795 nm.

The measurements of the saturation characteristics of the amplifier were carried out using an in-home built Bi-doped fiber laser operating at 1680 nm as a signal source. The power of the input signal was varied by an optical attenuator.

**Figure 4:**
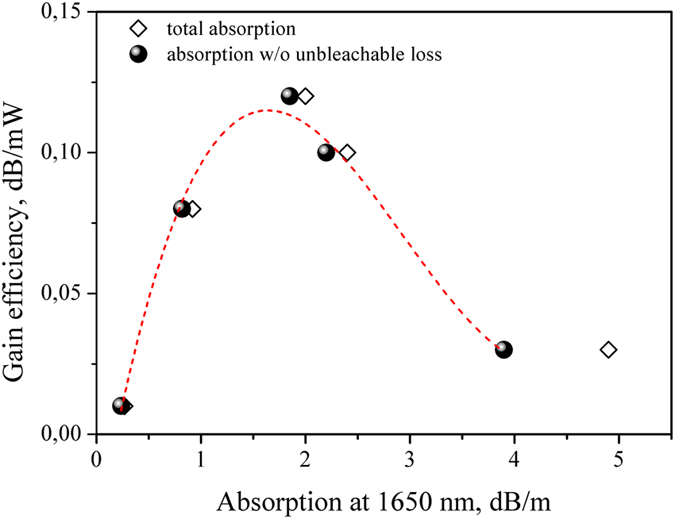
Gain efficiency at 1700 nm as a function of the total absorption at the wavelength of 1650 nm (rhombus) and the absorption of BACs (balls).

A family of optical fiber amplifiers utilizing the Bi-doped fibers presented in Table 1 was developed. In each case, the length of the active fiber was experimentally chosen to be close to optimal, i.e. the required fiber length for which gain is maximized at given pump and signal powers. The gain efficiency of the BDFA was measured at the wavelength 1700 nm. As a result, the experimental dependence of gain efficiency versus absorption was obtained (Fig. 4). Each point in the graph corresponds to a particular fiber from Table 1. The Y-coordinate of each point in the graph is equal to the maximal gain efficiency which was achieved for the fiber. The X-coordinate is equal to either the total loss at the wavelength of 1650 (rhombus) or the bleachable loss at the same wavelength (balls). Figure 4 shows that the gain efficiency can be raised by increasing the bismuth concentration while the total absorption at the wavelength 1650 nm is still less than 1.5 dB/m. At a higher concentration the gain efficiency is dramatically decreased. So, the optimum total concentration of bismuth is close to 0.015–0.02 weight% (Table 1). We suppose that the optimum bismuth concentration will be raised by the optimization of technological process.

**Figure 5:**
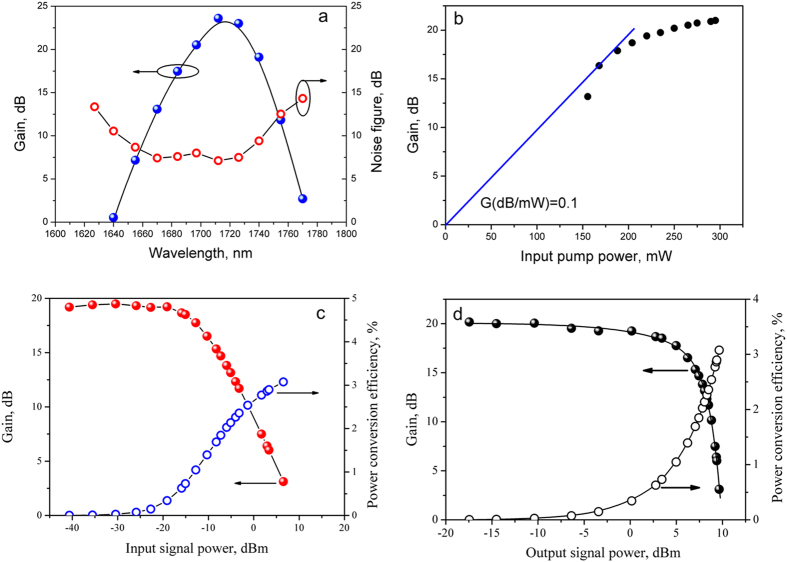
Characteristics of the BDFA: (**a**) gain and noise figure as a function of wavelength; (**b**) gain at 1700 nm versus pump power at 1550 nm; (**c**,**d**) gain at 1680 nm and power conversion efficiency versus input signal power, output signal power, respectively.

Taking into account the obtained results, we utilized Fiber #227 for the construction of the BDFA whose characteristics were then studied in detail. The length of the active fiber was 50 m. Figure 5(a) represents spectral dependencies of the gain and the noise figure of the BDFA (Table 1). These dependencies were obtained under bidirectional pumping with the total power of 300 mW. The input signal power in each line was maintained at lower than −20 dBm. This input signal power did not lead to saturation of the amplifier, i.e. the amplifier operated in a small signal gain regime. Therefore, the input signal power does not noticeably affect the gain for the measured wavelengths. It is seen that the maximum gain reached is 23 dB at the wavelength 1710 nm. The 3-dB width of the gain spectrum is equal to 40 nm. The asymmetric shape of the spectrum is caused by WDMs insertion loss in the long-wavelength region. It can potentially be improved by a proper WDM selection. The minimum noise figure is about 7 dB in a spectral range of 1670–1730 nm.

Figure 5(b) shows the BDFA gain with respect to the input pump power at the wavelength 1550 nm. In this case, the co-directional pump power (150 mW) was fixed whereas the contra-directional pump power was varied from 0 to 150 mW. The maximum gain coefficient of the BDFA was 0.1 dB/mW. Saturation characteristics of the BDFA under 300-mW pumping are shown in Fig. 5(c,d). The conversion efficiency is defined as (S_out_ − S_in_)/P_in_, where S_in_, S_out_ and P_in_, are a launched signal power, an output signal power, and a launched pump power, respectively. The input and output signal powers at 3 dB gain compression were −13 and +5 dBm, respectively, corresponding to a pump to signal conversion efficiency of ~1%.

We summarized the main parameters of the optical amplifier developed in present work and the results of other available (to our knowledge) fiber amplifiers for this spectral region (Table 2). It is seen, that most characteristics of the BDFA are comparable to the ones of TDFA based on fluoride glass fiber. It should be noted that the BDFA has a wider 3-dB gain bandwidth than both TDFAs. The comparison of the BDFA with the TDFA based on a silica glass fiber is hard to accomplish because of the lack of sufficient published data (in particular, pump power). However, a gain per meter of the Tm-doped fibers is higher than the ones of the Bi-doped fibers. As a result, several tens meters of a fiber are commonly used to realize Bi-doped fiber amplifiers whereas lengths of Tm-doped fiber amplifiers are significantly shorter.

In conclusion, the first bismuth-doped fiber amplifier (BDFA) for a spectral range of 1640–1770 nm has been developed. The BDFA pumped with commercially available laser diodes using a bidirectional configuration demonstrates the maximum gain 23 dB at 1710 nm, the 3-dB bandwidth 40 nm, the gain efficiency 0.1 dB/mW and the minimum noise figure ~7 dB. The comparison of characteristics between BDFA and TDFAs operating in the same spectral region is presented. Possible ways to improve the BDFA characteristics are to optimize the fiber fabrication process and chemical glass composition.

## Methods

The input and amplified signals were collected and recorded using an optical spectrum analyzer (OSA) HP 70950B for a wavelength range of 1500–1700 nm and a grating monochromator equipped with an InGaAs photodetector for a wavelength range of 1600–2000 nm. The output pump powers provided by laser diodes at 1550 nm were measured by an Ophir FS power meter. All measurements were made at room temperature.

## Additional Information

**How to cite this article**: Firstov, S. V. *et al*. A 23-dB bismuth-doped optical fiber amplifier for a 1700-nm band. *Sci. Rep.* **6**, 28939; doi: 10.1038/srep28939 (2016).

## Figures and Tables

**Table 1:** Characteristics of bismuth-doped high-germania fibers.

#	Total Bi concentration, 10^−3^ wt.%	Total absorption at 1650 nm, dB/m	Unbleachable loss at 1650 nm, dB/m
226	1.5	0.27	~0.05
232	4.5	0.92	0.11
217	15	2.0	0.15
227	18	2.4	0.2
218	40	4.9	1

**Table 2:** Main characteristics of the BDFA and the TDFA operating in near 1700 nm.

Parameter	BDFA (~50GeO_2_-50SiO_2_) [present work]	TDFA (silica-based glass)^24^	TDFA (fluoride glass)^23^
Pump power, mW	300	–	277
Small signal gain, dB	23	29	22.5
Gain coefficient, dB/mW	0.1	–	~0.08
Noise Figure, dB	~7	6.5	6.2
3 dB gain bandwidth, dB	40	15	31
Peak wavelength, nm	1710	1690	1695
Pumping source, nm	LD 1550	Fiber Laser 1560	LD 1210
